# Increasing number of unmanaged distressing symptoms in people living with dementia associated with family carer burden and distress following an unplanned hospital admission: A longitudinal cohort study

**DOI:** 10.1177/26323524261440961

**Published:** 2026-05-07

**Authors:** Sophie Crawley, Victoria Vickerstaff, Clare Ellis-Smith, Charlotte Kenten, Emel Yorganci, Catherine J. Evans, Elizabeth L. Sampson, Nuriye Kupeli

**Affiliations:** 1 Marie Curie Palliative Care Research Department, Division of Psychiatry, University College London, UK; 2 Centre for Evaluation and Methods, Wolfson Institute of Population Health, Queen Mary University of London, UK; 3 Cicely Saunders Institute of Palliative Care, Policy & Rehabilitation, Florence Nightingale Faculty of Nursing, King’s College London, UK; 4 Centre for Psychiatry and Mental Health, Wolfson Institute of Population Health, Queen Mary University of London, UK; 5 Sussex Community NHS Foundation Trust, Brighton General Hospital, UK; 6 Department of Psychological Medicine, Royal London Hospital, East London NHS Foundation Trust, UK

**Keywords:** dementia, end of life, family carers, symptoms, hospital, longitudinal

## Abstract

**Background:**

People living with dementia (PLWD) often have high symptom burden, exacerbated by comorbidities and experience unplanned hospital admissions that increase towards the end of life. Unmanaged symptoms and unplanned hospital admissions are distressing for the person living with dementia and family carers and expensive for the healthcare system.

**Objective:**

To explore the relationship between unmanaged symptoms of PLWD following an unplanned hospital admission and carer health and wellbeing outcomes over time.

**Design:**

Prospective longitudinal cohort study.

**Methods:**

Physical, psychosocial and spiritual symptoms of the PLWD and carer burden, distress and pre-death grief were assessed over 12 months following an unplanned hospital admission. Descriptive statistics and multilevel linear regression analyses were used to examine the relationships between PLWD unmanaged symptoms and carer outcomes.

**Results:**

A total of 51 carers of PLWD with a mean age of 59.6 participated. Carer burden was significantly associated with increasing PLWD unmanaged symptoms (coefficient = 0.07, 95% CI [0.03, 0.12], *p* = 0.002) and agitation (coefficient = 0.08, 95%CI [0.02, 0.14], *p* = 0.009). Carer psychological distress showed a positive trend in its association with increasing PLWD unmanaged symptoms (coefficient = 0.07, 95% CI [−0.00, 0.13], *p* = 0.059) and agitation (coefficient = 0.06, 95% CI [−0.01, 0.14], *p* = 0.090), but both analyses did not reach statistical significance. Carer pre-death grief was not associated with PLWD symptoms or agitation.

**Conclusion:**

Identifying and addressing symptoms in PLWD following an unplanned hospital admission is essential in enhancing the wellbeing of PLWD and carers. Hospital discharge planning should incorporate carer perspectives, provide support for carer burden and distress, and ensure continuity of care to reduce symptoms and improve outcomes for both PLWD and their carers.

## Introduction

Dementia is a life-limiting condition. A third of people over the age of 65 will die with dementia.^1^ The number of people with dementia expected to die with serious health-related suffering is projected to quadruple.^2^ Many people living with dementia (PLWD) have other age-related comorbidities^3^ often leading to high symptom burden and complex care needs. Unplanned hospital admissions are common for PLWD, especially in the last 6–12 months of life^4^ with factors such as comorbidities and neuropsychiatric symptoms found to increase the risk of hospitalisation^5^ and readmission to hospital.^6^

Unplanned hospital admissions are significant events, typically occurring during a period of acute crisis, which can be challenging for PLWD and their family carers.^7^ Increasing symptoms and difficulties in communication can be difficult to assess as dementia progresses, leading to poorer quality of life for the PLWD and higher distress for family carers.^8^ Links exist between behavioural and psychological symptoms of dementia (BPSD); namely depression, agitation and apathy, and carer wellbeing.^9–12^ However, the association between symptoms experienced by PLWD following an unplanned hospital admission and the impact an unplanned hospital admission has on the health and wellbeing of family carers has not been explored. This represents an important gap in the literature, as hospital admissions offer a valuable opportunity for re-engagement with services, comprehensive reassessment of symptoms and the implementation of appropriate interventions. Family carers play a pivotal role in recognising and addressing unmanaged symptoms in PLWD, and their capacity to do so may be shaped by their own health and wellbeing.^13,14^

Our aim was to explore the relationship between physical, psychosocial and spiritual symptoms presented by PLWD following an unplanned hospital admission and the experience of distress, burden and pre-death grief in family carers (referred to as carers from hereon).

## Methods

A longitudinal, prospective cohort study exploring the physical, psychosocial and spiritual symptoms of PLWD and the experiences of their carers following an unplanned hospital admission reported using the STROBE cohort guidelines.^15^ This study is from the Empowering Better End-of-Life Dementia Care (EMBED-Care) Programme, a multi-workstream research programme designed to create a step-change in the provision of palliative dementia care and improve end of life care experiences for PLWD and their carers.^16^

### Participants/recruitment

PLWD (aged ⩾65, with formal dementia diagnosis) and capacity to consent or had a personal consultee and their carers (aged ⩾18 providing any type of support) were recruited during unplanned hospital admissions from four NHS sites in South-East England between 2021 and 2024. This sample of PLWD was selected because, based on the existing literature described above, they are likely to be in the last 6–12 months of life.^4^ Exclusion criteria for PLWD was those who did not have a formal diagnosis of dementia, were unable to respond to study assessments and did not have a proxy respondent and those who were unable to communicate in English. Exclusion criteria for carers were those who were ⩽18 years, unable to communicate in English, and did not have the capacity to provide informed written consent. Eligible carers participated only if the PLWD’s assessments were completed. Assessments occurred every 4 months over 12 months or until the PLWD died. Data collection took place between January 2021 and February 2024, inclusive of follow-ups. Because of COVID-19 restrictions, data collection took place either by telephone or video.

### Measures

We collected data on the age, gender and ethnicity of both PLWD and their carers. For carers, additional data were collected on marital status, highest educational level, current employment status and relationship to the PLWD. For PLWD, we collected information on the type and severity of dementia and place of residence. Measures used to assess symptoms of PLWD, and those used to assess the carers’ health and wellbeing, are detailed in Supplemental Information.

Symptoms of the PLWD were assessed using the proxy-completed version of the Integrated Palliative Care Outcome Scale for Dementia (IPOS-Dem),^17^ an outcome measure to assess distressing physical symptoms and emotional, social and spiritual concerns of the PLWD, based on knowledge and observation of the PLWD. Higher scores indicate greater distress caused by symptoms. The Neuropsychiatric Inventory Questionnaire (NPI-Q)^18^ assessed agitation for the PLWD.

Carer burden, distress and pre-death grief were measured with the 12-item Zarit Burden Interview (ZBI-12),^19^ the Kessler Psychological Distress Scale (K-10)^20^ and the Caregiver Grief Questionnaire (CGQ),^21^ respectively. Higher scores indicate higher reported burden, distress and pre-death grief. Description of measures provided in Supplemental Table 1.

### Statistical analysis

Sociodemographic data were summarised using the mean (standard deviation, SD) and the median (interquartile range, IQR) as appropriate. Categorical data were presented as counts (percentages). Multilevel linear regression analyses were conducted to assess associations between carer outcomes (burden, distress and pre-death grief) and the number of unmanaged symptoms for the PLWD (IPOS-Dem)^17^ in model 1, and the presence of agitation (NPI-Q)^18^ in model 2. All data collected over the follow-up were included; these models accounted for the repeated measurements over the follow-up by incorporating random effects to adjust for correlations within-individuals. Models were adjusted for two potential confounders: relationship to the PLWD (child vs other) and living arrangement (care home vs at home). Because of the limited sample size, we did not adjust for additional variables to avoid overfitting the model. Coefficients (unstandardised), 95% confidence intervals (CIs) and *p* values were reported. Statistical analysis was performed in Stata (version 18), with statistical significance defined as a *p* value <0.05. Analyses were considered exploratory, and no adjustments were made for multiple comparisons. The sample size was determined based on a minimum of 10 cases per predictor variable to ensure stable coefficient estimates and minimise the risk of overfitting.^22^

## Results

Carers (*n* = 51) were female (67%) with a mean age of 60 years (SD = 12.8), mainly caring for a parent living with dementia (73%), with one carer for each PLWD taking part. PLWD were predominantly female (67%) with a mean age of 85 years (SD = 6.6), with moderate-to-severe dementia (84%), and were living at home (40%) or care homes/sheltered accommodation (60%). Sample characteristics for carers and PLWD are provided in Supplemental Table 2.

Baseline mean scores for carer outcomes are provided in Supplemental Table 3. Carers were categorised as caring for a PLWD with a low number of unmanaged symptoms (0–4 symptoms as assessed by the IPOS-Dem) and PLWD with a high number of unmanaged symptoms (⩾5 symptoms as assessed by the IPOS-Dem). Supplemental Table 4 provides the mean scores for the two groups.

Carer burden mean scores were higher for those caring for a PLWD experiencing high unmanaged symptoms (ZBI-12 *M* = 23.2 (SD = 10.0)) compared with those caring for someone experiencing low unmanaged symptoms (ZBI-12 *M* = 22.6 (SD = 12.2)). Conversely, carer pre-death grief and burden mean scores were higher in those caring for someone experiencing low unmanaged symptoms (CGQ *M* = 42.5 (SD = 7.0) and K-10 *M* = 22.9 (SD = 8.2)) compared with those caring for someone experiencing high unmanaged symptoms (CGQ *M* = 41.4 (SD = 10.5) and K-10 *M* = 21.9 (SD = 7.7)).

Carers were categorised as caring for a PLWD who showed signs of agitation or not, as assessed using the NPI-Q. Supplemental Table 5 provides the mean scores for the two groups. Carer burden and distress mean scores were higher in those caring for someone presenting with agitation (ZBI-12 *M* = 22.9 (SD = 10.6) and K-10 *M* = 22.4 (SD = 8.1)) compared with those not experiencing agitation (ZBI-12 *M* = 19.1 (SD = 9.4) and K-10 *M* = 20.5 (SD = 6.7)), while carer pre-death grief mean scores were higher for those caring for not experiencing agitation (CGQ *M* = 44.8 (SD = 7.1) vs *M* = 39.3 (SD = 10.7)).

Carer burden (ZBI-12) was significantly associated with increasing unmanaged symptoms experienced by PLWD in model 1 and increasing agitation, in model 2, and carer distress (K-10), while not statistically significant, showed a positive trend in its association with increasing unmanaged symptoms and agitation in models 1 and 2, respectively. Both models showed no evidence of an association between carer pre-death grief (CGQ) and unmanaged symptoms or agitation. Table 1 provides the results of the two models.

**Table 1.** Relationship between carer outcomes with unmanaged symptoms presented by PLWD as assessed using IPOS-Dem (model 1) and agitation as assessed using NPI-Q (model 2).

	Number of observations	Coefficient	95% CI	*p* Value
Model 1: Unmanaged symptoms (IPOS-DEM)				
CGQ total score	67	−0.02	[−0.09, 0.05]	0.610
ZBI-12 total score	123	0.07	[0.03, 0.12]	0.002
K-10 total score	127	0.07	[−0.00, 0.13]	0.059
Model 2: Agitation (NPI-Q)				
CGQ total score	65	−0.03	[−0.09, 0.03]	0.349
ZBI-12 total score	121	0.08	[0.02, 0.14]	0.009
K-10 total score	125	0.06	[−0.01, 0.14]	0.090

CGQ: Carer Grief Questionnaire; IPOS-Dem: Integrated Palliative care Outcome Scale for Dementia; K-10: Kessler Psychological Distress Scale; NPI-Q: Neuropsychiatric Inventory Questionnaire; ZBI-12: Zarit Burden Interview.

## Discussion

This prospective longitudinal cohort study highlights the relationship between experiences of the PLWD and carer outcomes by demonstrating that a higher number of common symptoms, such as pain, problems sleeping, difficulty communicating, poor appetite and agitation in PLWD, contribute to burden and distress in carers. This aligns with prior research showing that the complexity and severity of unmanaged symptoms directly increase carer burden.^23^ Similarly, carers of PLWD with agitation exhibited significantly higher burden and psychological distress compared to carers of individuals without agitation, reinforcing agitation as a particularly distressing symptom for carers.^24^ Importantly, the impact on carer outcomes remained regardless of different relationship types (adult child or other) and living arrangement (care home or at home). This suggests that symptom burden is a critical factor influencing carer wellbeing across diverse caregiving contexts, highlighting the importance of symptom management in reducing carer strain. Most importantly, the symptoms of the PLWD in our sample may have emerged during or following the unplanned hospital admission, underscoring the need for better continued symptom assessment and management.

As opposed to limited evidence from previous studies, our findings show that carer pre-death grief scores were higher among carers of PLWD who had fewer unmanaged symptoms and in those caring for individuals without agitation. This finding appears counterintuitive, as previous research suggests that greater behavioural and psychological symptoms in PLWD are typically associated with higher levels of pre-death grief, although this conclusion is based on limited evidence.^11^ While definitive conclusions cannot yet be drawn, it is possible that pre-death grief experiences are influenced by different mechanisms than those underlying burden and distress. In particular, pre-death grief may be shaped more by psychological and relational factors, such as perceived loss of connection, changing roles, awareness of impending decline and difficulties in knowing how to prepare for the end of life, rather than by the severity of dementia symptoms alone.^11,25^ These findings underscore the complexity of grief in dementia caregiving and highlight the importance of providing nuanced support that addresses both emotional and existential dimensions of the caregiving experience.

A potential limitation of the study is that participants were recruited during the COVID-19 pandemic, which may have introduced additional uncertainty and variability in care experiences. However, we collected data over the full 12-month study period (where available), providing a broader longitudinal perspective. In addition, unplanned hospital admissions are not uncommon for PLWD (prior to the pandemic, hospitalisation rates for PLWD ranged from 0.37 to 1.26 a year).^26^ As most PLWD experience an unplanned hospital admission in the last year of life, our study may have identified a time of greater need, and where carers may require additional support from their peers and formal services.

Recruitment was impacted by the COVID-19 pandemic restrictions, including delays in hospital-based recruitment and restrictions that prevented carers of PLWD in care homes from visiting PLWD. Thus, data collection was conducted remotely once the PLWD had been discharged from the hospital, which meant that in-person observations of the PLWD were not possible. To ensure consistency and completeness, structured case report forms were used, and data were collected by trained researchers with experience working with PLWD and their carers. However, while virtual data collection in dementia research has been shown to be feasible,^27^ we acknowledge two limitations. First, the combination of remote data collection and proxy-respondent ratings for assessments of unmanaged symptoms experienced by PLWD may have influenced our findings. Second, as data collection began after the PLWD had left the hospital, we were unable to capture information on symptom onset, making it difficult to determine if the unmanaged symptoms captured during assessment were related to the unplanned hospital admission. However, despite these challenges, the sample we recruited, primarily female carers supporting parents with moderate-to-severe dementia, and predominantly older females living with dementia, aligns with established family carer demographics^28^ and known characteristics of PLWD,^29^ thereby increasing the generalisability of our findings. Finally, while we did not conduct a formal sample size calculation for the cohort of carers, and the overall sample was modest, the study was sufficiently powered for the planned regression analyses.^22^

Given the significant reliance on carers,^30^ identifying ways to reduce carer distress and burden may have a positive impact on both PLWD and their carers, enhancing carers’ ability to continue providing care. Although we were unable to include all factors that may contribute to carer burden and distress, such as social support,^31^ due to limited statistical power, our findings highlight the need for comprehensive assessment and management of symptoms in PLWD, particularly agitation, to improve their quality of life and mitigate carer burden and distress. Integrating carer support into discharge planning and post-hospital care may improve outcomes for both PLWD and their carers.^7^ However, for this approach to be effective, healthcare systems must be structured to deliver accessible training and education for carers, strengthen respite provision and offer support in managing BPSD, ensuring that carers are recognised, supported and embedded as integral partners in care.^32–34^

Future research should prioritise the development and evaluation of targeted interventions that address the symptoms most strongly associated with carer burden and distress, such as agitation. Qualitative studies could provide deeper insights into the nuanced experiences of pre-death grief among carers, helping to shape more holistic and responsive support strategies.

## Conclusion

In conclusion, addressing unmanaged symptoms and agitation in PLWD is likely to play an important role in reducing carer burden and distress. Tailored interventions that consider the distinct needs of family carers following unplanned hospital admissions are needed.

## Supplemental Material

sj-docx-1-pcr-10.1177_26323524261440961 – Supplemental material for Increasing number of unmanaged distressing symptoms in people living with dementia associated with family carer burden and distress following an unplanned hospital admission: A longitudinal cohort study Supplemental material, sj-docx-1-pcr-10.1177_26323524261440961 for Increasing number of unmanaged distressing symptoms in people living with dementia associated with family carer burden and distress following an unplanned hospital admission: A longitudinal cohort study by Sophie Crawley, Victoria Vickerstaff, Clare Ellis-Smith, Charlotte Kenten, Emel Yorganci, Catherine J. Evans, Elizabeth L. Sampson and Nuriye Kupeli in Palliative Care and Social Practice

sj-docx-2-pcr-10.1177_26323524261440961 – Supplemental material for Increasing number of unmanaged distressing symptoms in people living with dementia associated with family carer burden and distress following an unplanned hospital admission: A longitudinal cohort study Supplemental material, sj-docx-2-pcr-10.1177_26323524261440961 for Increasing number of unmanaged distressing symptoms in people living with dementia associated with family carer burden and distress following an unplanned hospital admission: A longitudinal cohort study by Sophie Crawley, Victoria Vickerstaff, Clare Ellis-Smith, Charlotte Kenten, Emel Yorganci, Catherine J. Evans, Elizabeth L. Sampson and Nuriye Kupeli in Palliative Care and Social Practice
