# Supplementary material for: Increasing number of unmanaged distressing symptoms in people living with dementia associated with family carer burden and distress following an unplanned hospital admission: A longitudinal cohort study
Source: Palliat Care Soc Pract. 2026 May 7;20:26323524261440961. doi: 10.1177/26323524261440961 (PMC13167373; doi:10.1177/26323524261440961)
Supplement: sj-docx-2-pcr-10.1177_26323524261440961 – Supplemental material for Increasing number of unmanaged distressing symptoms in people living with dementia associated with family carer burden and distress following an unplanned hospital admission: A longitudinal cohort study [file sj-docx-2-pcr-10.1177_26323524261440961.docx]

# Supplementary information

**Table 1:** Description of the measures used to assess symptoms experienced by PLWD and measures of carer health and wellbeing

| **Measure** | **Description** |
| --- | --- |
| **PLWD measures** | |
| Clinical Dementia Rating (CDR) scale^1^ | Measure of dementia severity that uses a five-point Likert scale to assess the severity of signs and symptoms in six areas of functioning including memory, orientation, judgment, and problem solving, community affairs/involvement, home life and hobbies, and personal care. Scores indicate no dementia (0), very mild dementia (0.5), mild dementia (1), moderate dementia (2) and severe dementia (3). It has been shown to be a reliable and valid global measure of dementia severity^1^. |
| Integrated Palliative care Outcome Scale for Dementia (IPOS-Dem^2^) | Measure of physical, psychosocial and spiritual symptoms designed to support assessment of common symptoms and problems such as pain, nausea and poor appetite experienced by this population^2^. It consists of 11 questions with a number of different response scales (e.g., “not at all” to “always”). Three versions of the measure available to reflect whether the person completing the measure is the PLWD, family carer or formal carer/health care professional. Only one version of the tool is completed. Higher scores indicate higher distress caused by common symptoms. For the purpose of this study, we categorised PLWD as those experiencing low unmet symptoms (those who scored moderately, severely or overwhelmingly for less than four symptoms) and those with high unmet symptoms (those who scored moderately, severely or overwhelmingly for five or more symptoms). |
| Neuropsychiatric Inventory Questionnaire (NPI-Q^3^) | Measure of behavioural and psychological symptoms commonly observed in PLWD including psychosis, mood disturbances, agitation, personality changes, pacing, wandering, and appetite disturbances. The NPI-Q is a brief and validated version of the interview-based NPI^4^. For the purpose of this study we used this measure for assessing agitation and categorised PLWD as presenting with agitation or not. |
| **Carer measures** | |
| Caregiver Grief Questionnaire (CGQ^5^) | Measure of grief experienced by carers before the person they are caring for has died (pre-death grief) comprising two domains assessing relational deprivation (six items) and emotional pain (five items). Items include “I miss so many of the activities we used to share” and “I lost someone who really knew me well”. Each item is assessed on a five-point Likert scale (“strongly agree” to “strongly disagree”) with higher scores indicating more grief. This relatively new measure has demonstrated promising psychometric properties including structural validity and internal consistency when assessed with family carers of PLWD^5^. |
| Kessler Psychological Distress Scale (K-10^6^) | Global measure of distress based on questions about anxiety and depressive symptoms experienced in the last four weeks. It consists of 10 items measured on a five-point Likert scale (“none of the time” to “all of the time”) with higher scores indicating higher levels of distress. The K-10 has been found to have good internal consistency and sensitivity and specificity of over 65% for detecting depression and anxiety in community samples^7^. |
| Zarit Burden Interview (ZBI-12^8^) | A 12-item self-report measure of carer burden in dementia adapted from the 22-item version of the ZBI^9^. Items require carers to reflect on how they feel when they are caring for the person and includes items such as “Do you feel that because of the time you spend with your relative that you don't have enough time for yourself?” Each item is rated on afive5-point Likert scale ranging from “never” to “nearly always” with higher scores indicating higher levels of burden. For the purpose of this study we will use a shortened 12-item version of the ZBI^8^ which has been shown to have good psychometric properties^10^. |

**Table 2:** PLWD and carer demographics

| **Carer variables** | **Baseline (n=51)** |
| --- | --- |
| *Age* M(SD[range]) | 59.6 (12.8[24-89]) |
| *Gender, n (%)*  Female  Male | 34 (66.7)  17 (33.3) |
| *Ethnicity, n (%)*  White British  Caribbean  Indian  African  White & Asian  Other/prefer not to say | 40 (78.4)  2 (3.9)  2 (3.9)  1 (2.0)  1 (2.0)  5 (9.9) |
| *Marital status, n (%)*  Married/ civil partnership  Single  Divorced  Co-habiting  Other (partner, long term relationship) | 31 (60.8)  8 (15.7)  5 (9.8)  4 (7.8)  3 (5.9) |
| *Education, n (%)*  NVQ (or equivalent)  Degree  A level (or equivalent)  GCSE (or equivalent)  Higher degree  School Leaving Certificate | 12 (23.5)  11 (21.6)  11 (21.6)  9 (17.6)  6 (11.8)  2 (2.9) |
| *Employment, n (%)*  In paid employment  Retired  Not working | 22 (43.1)  26 (31.4)  13 (25.4) |
| *Live with PLWD, n (%)*  No  Yes | 35 (68.6)  16 (31.4) |
| *Relationship with PLWD, n (%)*  Adult child  Spouse/partner  Sibling  Other (Daughter-in-law, Granddaughter, Stepdaughter) | 37 (72.5)  9 (17.6)  1 (2.0)  4 (7.8) |
| *Religion, n (%)*  Church of England  Catholic  No specific religious background  Muslim  Hindu  Other/Prefer not to say | 24 (47.1)  9 (17.6)  9 (17.6)  3 (5.9)  1 (2.0)  5 (9.8) |
| **PLWD variables** | |
| *Age* M(SD[range]) | 85.2 (6.6[70-97]) |
| *Gender, n (%)*  Female  Male | 34 (66.7)  17 (33.3) |
| *Dementia severity CDR, n (%)*  Severe  Moderate  Mild | 22 (43.1)  21 (41.2)  8 (15.7) |
| *Dementia diagnosis, n (%)*  Alzheimer’s disease  Vascular dementia  Mixed dementia  Dementia with lewy bodies  Frontotemporal dementia  Unknown/other | 15 (29.4)  14 (27.5)  6 (11.8)  2 (3.9)  1 (2.0)  12 (23.5) |
| *Place of residence, n (%)*  At home  Care home/sheltered accommodation | 20 (40%)  30 (60%) |

***Note.*** M=Mean, SD=Standard Deviation, NVQ=National Vocational Qualification, GCSE=General Certificate of Secondary Education

**Table 3:** Carer study entry scores

|  | **N** | **Mean (SD)** | **Median** | **IQR** |
| --- | --- | --- | --- | --- |
| CGQ total score | 46 | 41.0 (10.0) | 43 | (34, 49) |
| ZBI-12 total score | 50 | 22.2 (10.7) | 22.5 | (13, 30) |
| K-10 total score | 51 | 22.3 (7.8) | 22 | (16, 26) |

***Note.*** SD=Standard Deviation; IQR=Interquartile Range; CGQ=Caregiver Grief Questionnaire; ZBI-12=Zarit Burden Interview-12; K-10=Kessler Psychological Distress Scale-10

**Table 4:** Carer study entry scores by whether PLWD had low (0-4 symptoms) or high (**≥**5 symptoms) number of unmet symptoms as assessed using the IPOS-Dem

|  | **Number of unmet symptoms** | | | | | |
| --- | --- | --- | --- | --- | --- | --- |
|  | **Low (0-4)** | | | **High (≥5)** | | |
|  | **Mean (SD)** | **Median** | **IQR** | **Mean (SD)** | **Median** | **IQR** |
| CGQ total score | 42.5  (7.0) | 41 | (37.5,48) | 41.4 (10.5) | 44 | (33, 51) |
| ZBI-12 total score | 22.6 (12.2) | 21.5 | (16,29) | 23.2 (10.0) | 27 | (17, 30) |
| K-10 total score | 22.9  (8.2) | 22.5 | (16,26) | 21.9  (7.7) | 21.5 | (16,25) |

***Note.*** IPOS-Dem= Integrated Palliative care Outcome Scale for Dementia; SD=Standard Deviation; IQR=Interquartile Range; CGQ=Caregiver Grief Questionnaire; ZBI-12=Zarit Burden Interview-12; K-10=Kessler Psychological Distress Scale-10

**Table 5:** Carer study entry scores by whether the PLWD showed signs of agitation or not as assessed using the NPI-Q

|  | **Agitation** | | | | | |
| --- | --- | --- | --- | --- | --- | --- |
|  | **No** | | | **Yes** | | |
|  | **Mean (SD)** | **Median** | **IQR** | **Mean (SD)** | **Median** | **IQR** |
| CGQ total score | 44.8  (7.1) | 43 | (41, 52) | 39.3  (10.7) | 42 | (33, 48) |
| ZBI-12 total score | 19.1  (9.4) | 17 | (13,24) | 22.9  (10.6) | 27 | (12, 30) |
| K-10 total score | 20.5  (6.7) | 19.5 | (16, 24) | 22.4  (8.1) | 22 | (16, 26.5) |

***Note.*** NPI-Q=Neuropsychiatric Inventory Questionnaire; SD=Standard Deviation; IQR=Interquartile Range; CGQ=Caregiver Grief Questionnaire; ZBI-12=Zarit Burden Interview-12; K-10=Kessler Psychological Distress Scale-10

## References

1. Morris JC. Clinical dementia rating: a reliable and valid diagnostic and staging measure for dementia of the Alzheimer type. *International psychogeriatrics* 1997; 9: 173-176.

2. Ellis-Smith C, Evans CJ, Murtagh FE, et al. Development of a caregiver-reported measure to support systematic assessment of people with dementia in long-term care: The Integrated Palliative care Outcome Scale for Dementia. *Palliative medicine* 2017; 31: 651-660.

3. Kaufer DI, Cummings JL, Ketchel P, et al. Validation of the NPI-Q, a brief clinical form of the Neuropsychiatric Inventory. *The Journal of neuropsychiatry and clinical neurosciences* 2000; 12: 233-239.

4. Cummings JL. The neuropsychiatric inventory: Assessing psychopathology in dementia patients. *Neurology* 1997; 48: S10-S16.

5. Cheng S-T, Ma DY and Lam LC. A brief measure of predeath grief in dementia caregivers: the Caregiver Grief Questionnaire. *International psychogeriatrics* 2019: 1-9.

6. Kessler RC, Andrews G, Colpe LJ, et al. Short screening scales to monitor population prevalences and trends in non-specific psychological distress. *Psychological medicine* 2002; 32: 959-976.

7. Andersen L, Grimsrud A, Myer L, et al. The psychometric properties of the K10 and K6 scales in screening for mood and anxiety disorders in the South African Stress and Health study. *International Journal of Methods in Psychiatric Research* 2011; 20: 215-223.

8. Bédard M, Molloy DW, Squire L, et al. The Zarit Burden Interview: a new short version and screening version. *The gerontologist* 2001; 41: 652-657.

9. Zarit SH, Todd PA and Zarit JM. Subjective burden of husbands and wives as caregivers: A longitudinal study. *The Gerontologist* 1986; 26: 260-266.

10. Higginson IJ, Gao W, Jackson D, et al. Short-form Zarit Caregiver Burden Interviews were valid in advanced conditions. *Journal of clinical epidemiology* 2010; 63: 535-542.
